# A novel technique for protecting staff during microlaryngoscopy procedures

**DOI:** 10.1017/S0022215121000037

**Published:** 2021-01-08

**Authors:** T Zoabi, O Ronen

**Affiliations:** 1 Department of Otolaryngology – Head and Neck Surgery, Saint Vincent De Paul Hospital, Nazareth, affiliated with Azrieli Faculty of Medicine, Bar Ilan University, Safed, Israel; 2 Department of Otolaryngology – Head and Neck Surgery, Galilee Medical Center, Nahariya, affiliated with Azrieli Faculty of Medicine, Bar Ilan University, Safed, Israel

**Keywords:** Otorhinolaryngologic Surgical Procedures, Equipment, Infection Control, SARS-CoV-2, 2019 Novel Coronavirus, COVID-19

## Abstract

**Objective:**

Microlaryngoscopy is an aerosol-generating procedure. This paper presents a novel approach for better protecting staff during microlaryngoscopy.

**Methods:**

A clear plastic microscope drape is attached to the objective lens. Instead of using the drape to cover the microscope, it is pulled down to cover the patient's head and torso. The holes designated for the binoculars of the microscope are used for the surgeon hands, forming protective clear plastic sleeves.

**Conclusion:**

The proposed technique, which is simple, relatively inexpensive and technically feasible for any hospital to perform during microlaryngoscopy procedures, can increase safety and minimise droplet and aerosol exposure in the operating theatre.

## Introduction

Coronavirus disease 2019 (Covid-19) has high transmissibility as it can spread via aerosol.^1^ The symptoms range from a mild cough to a unique acute respiratory distress syndrome. Some patients have been confirmed as asymptomatic.^2^ Since the outbreak of the coronavirus pandemic, many centres have narrowed down their elective surgical procedures, performing only urgent and emergent surgical procedures, including oncological diagnostic and therapeutic surgical procedures.

Patients with laryngeal findings suspected as malignant must undergo a diagnostic biopsy, usually as part of a suspension direct microlaryngoscopy procedure. Microlaryngoscopy is known to be an aerosol-generating procedure (AGP), and, with the high transmissibility of the virus,^3^ techniques are needed to maximise the safety of hospital staff. Attempting to obtain a biopsy in a clinic setting, using a fibre-optic endoscope with a working channel, puts the staff at a high risk of infection, especially in the absence of a practical way to prevent aerosol generated during the procedure from spreading within the clinic. We believe that obtaining the biopsy in an operating theatre setting is preferable, where it is possible to drape the anaesthetised patient, and the room is thoroughly cleaned between procedures.

Currently, there is little evidence or regulatory guidance to mitigate the intra-operative transmission of Covid-19 during microlaryngoscopy. Efforts have been made to screen patients prior to performing surgical procedures by using throat and nasal swabs for reverse transcription polymerase chain reaction testing; unfortunately, this method cannot ensure staff safety, given the significant false negative rate of this test, particularly during the first days of infection.^4^ Therefore, we propose a protocol used by our otolaryngology team to perform suspension direct microlaryngoscopy for patients with suspicious laryngeal findings.

This article presents a novel approach for better staff protection during microlaryngoscopy in the era of the Covid-19 pandemic. This technique is inexpensive, simple and ensures the functionality of the procedure. Moreover, it can be adopted for other similar AGPs that utilise a surgical microscope.

## Materials and methods

This study was approved by the institutional review board.

Prior to entering the operating theatre, the anaesthesiology team and nurses don personal protective equipment (PPE), consisting of an N95 mask, face shield, gloves and isolation gown. After the minimal necessary anaesthesia, the patient is placed in the supine position on the operating table. The anaesthesiology team administers anaesthesia and intubates using a transoral endotracheal cuffed tube guided by a GlideScope video laryngoscope (Verathon, Bothell, Washington, USA). After intubation has been carried out, the operating team, which donned PPE outside the operating theatre, can enter.

**Fig. 1.**
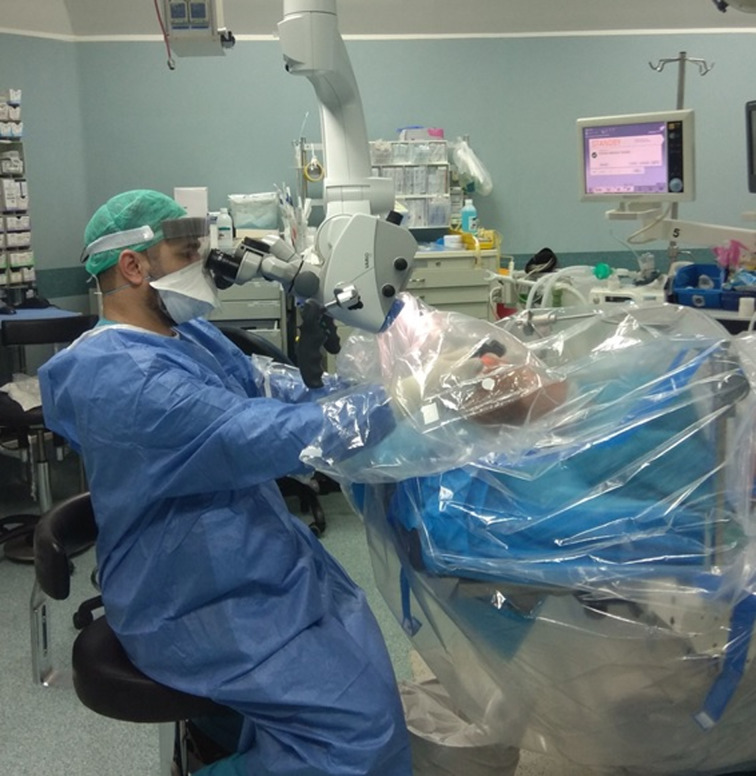
A clear plastic microscope drape is attached to its designated place on the objective lens; the drape is pulled down to cover the patient's head and torso, and is secured with tape. The surgeon's hands are inserted through the holes designated for the binoculars of the microscope, and secured with a tape at the level of the wrist.

**Fig. 2.**
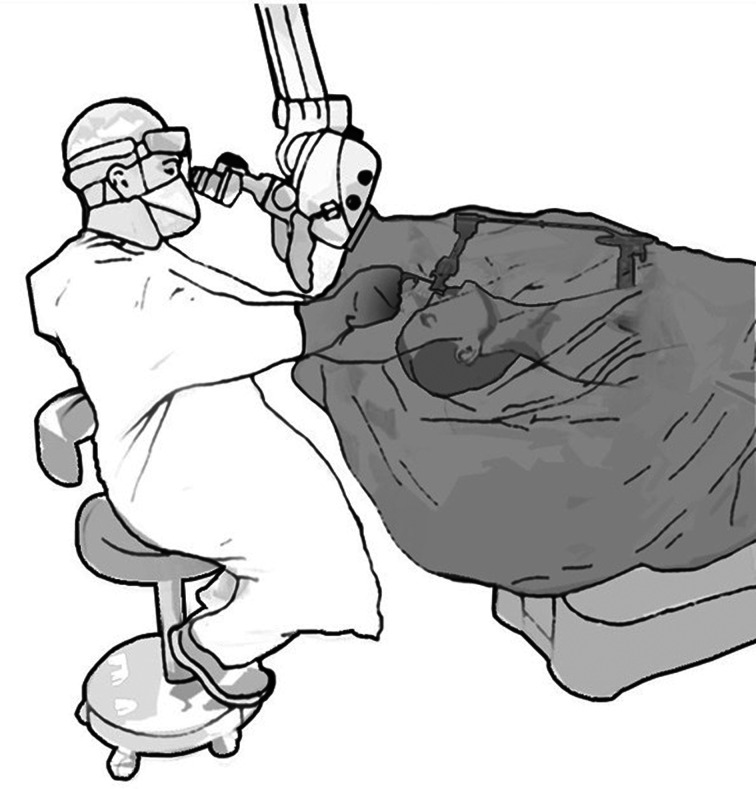
A simplified illustration of the novel approach of draping during microlaryngoscopy in the era of the coronavirus disease 2019 pandemic.

The laryngoscope, forceps, suction equipment, specimen collection container, pledgets and all other required instruments, are laid beside the patient's head. A clear plastic microscope drape is then attached to its designated place on the objective lens (Pharma Sept Medical Products, Caesarea, Israel). Instead of using the drape to cover the microscope, it is pulled down to cover the patient's head and torso and secured with tape, to maintain tightness. This creates a clear barrier between the surgeon and the operative field and instruments. The holes designated for the binoculars of the microscope are used for the surgeon's hands; these are secured tightly with tape at the level of the wrist, forming protective clear plastic sleeves, while providing proper reach to the instruments and the surgical field (Figures 1 and 2; see also the short video, available on *The Journal of Laryngology & Otology* website (Appendix 1).).

In this fashion, the surgical field is contained inside the clear plastic drapes, and the only openings are secured onto the gloved hands of the surgeon and the objective lens of the microscope. Thus, the risk of virion transmission via aerosol droplets is minimised, while still allowing for good visualisation and full range of motion within the operative field. The surgeon can now proceed with their hands underneath the drape, where the surgical field and all the instruments were placed prior to the draping. When the procedure ends, the drape is removed from the lens, pulled back and folded, and disposed of safely. The surgery team leaves the operating theatre to minimise staff exposure while the anaesthesiology team extubates the patient.

## Discussion

### Synopsis of key findings

Amidst the Covid-19 pandemic, many suspension direct microlaryngoscopy procedures required for the diagnosis of suspected laryngeal lesions are being postponed because of the fear of infecting the medical staff during this highly AGP. This risk can be reduced by using novel techniques that ensure the safety of the staff, such as the technique proposed above.

This protocol was first used on a patient with a history of laryngeal carcinoma who was treated with radiotherapy several years ago; the patient presented at follow up to our clinic with a new lesion on his right true vocal fold suspected as being malignant. Prior to conducting this protocol on a patient, we performed several simulations, assuring visibility of surgical site while keeping it contained.

Ultimately, this technique increases the safety of the hospital staff by shielding them from the aerosol generated during direct microlaryngoscopy procedures.

### Limitations

First, the surgery should also be carried out in a negative pressure operating theatre when possible. Second, the set-up allows for only a limited number of cold instruments to be placed under the cover during the procedures. Third, this technique may or may not allow for the use of a microdebrider or a laser, which we did not try. This might limit the spectrum of cases to only those that are amenable to cold instrument surgery.

## Conclusion

In the setting of the current global Covid-19 pandemic, with the highly contagious properties of the virus, which can be spread via contact, droplets and aerosol, it is imperative to seek new techniques to protect the hospital staff while they provide essential medical and surgical care for patients.

The technique proposed in this article is simple, relatively inexpensive and technically feasible for any hospital, allowing microlaryngoscopy procedures to be performed safely, while minimising droplet and aerosol exposure within the operating theatre.

## Appendix 1. Supplementary video material

A short video demonstrating the novel technique for protecting staff during microlaryngoscopy procedures is available online at https://drive.google.com/file/d/1Zc5wzyPD5g3Dm8Ql9MJ_ELMisLf3tjmw/view?usp=sharing. *The Journal of Laryngology & Otology* website, at https://doi.org/10.1017/S0022215121000037.
